# Harlequin fetus born from Consanguinity: A deleterious case report

**DOI:** 10.12669/pjms.35.5.916

**Published:** 2019

**Authors:** Joti Devnani, Ujalla Kumari, Zil-e-Rubab

**Affiliations:** 1 Joti Devnani, Ziauddin Medical College, Ziauddin University, Karachi, Pakistan; 2 Ujalla Kumari, Ziauddin Medical College, Ziauddin University, Karachi, Pakistan; 3 Zil-e-Rubab, MBBS, M-Phil, PhD, Ziauddin Medical College, Ziauddin University, Karachi, Pakistan

**Keywords:** *ABCA12* gene, Autosomal recessive, Consanguinity, Harlequin Ichthyosis

## Abstract

Harlequin Ichthyosis (HI) is a dreadful skin disorder with steady rise of cases with prolonged survival. Harlequin fetus follows an autosomal recessive pattern with the incidence of 1in 300,000 live births. In the succeeding case report, a male child was born with keratinized and kaleidoscopic diamond pattern of skin suggestive of HI. He was born at 36th week of gestation from consanguineous marriage. The newborn remained under extensive intensive care in a tertiary care unit where he breathed his last on 11th day after birth. Prenatal diagnosis and genetic counseling is of vital importance due to the association of *ABCA12* mutation with HI.

## INTRODUCTION

Harlequin ichthyosis is a rare and the most radical form of autosomal recessive disorder that affects the skin.^1^ The typical presentation is extensively thick scaly skin with red diamond shape fissures.^2^ Additional features of Harlequin ichthyosis are ectropion, eclabium, anonychia, polydectaly, syndectaly (fused fingers and toes), lack of development of external parts of nose and ears and decreased mobility and flexion of joints.^3^-^6^ The term harlequin originates from a clowning character in Italian theatre that looks like the harlequin baby.^7^ Mutation in *ABCA12* gene on chromosome number 2 is considered to be the main culprit for development of harlequin fetus. *ABCA12* is responsible for the formation of skin barrier consisting of lipid glucosylceramides in the lamellar granules.^8^,^9^ The lethal outcome of HI is due to development of sepsis, respiratory failure, hyperthermia and dehydration. This case study projected to report a case of HI in third infant born of a first cousin marriage who expired on 11^th^ day of birth.

## CASE REPORT

**Fig.1:**
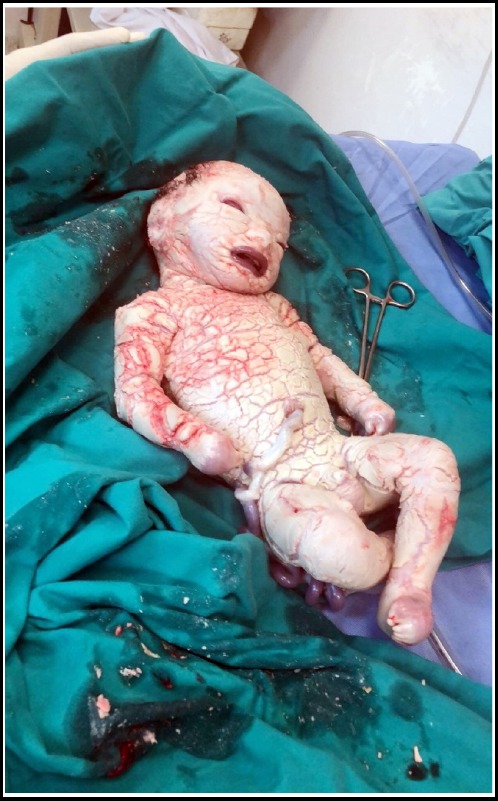
Harlequin Fetus at Birth

**Fig.2:**
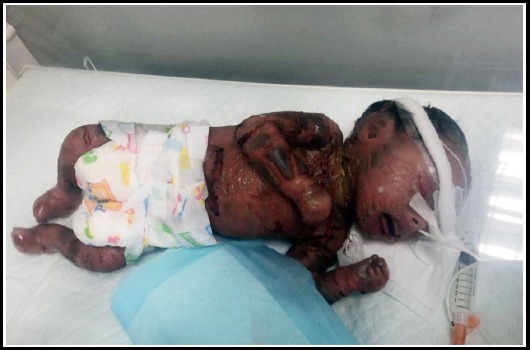
Shedding Off skin on Day 4 after Birth

A male child was born to a 28 years’ gravida 3 parity 2+0 by elective low segment caesarean section (ELSCS). She was brought to emergency room at gestational age of 36 weeks due to leaking (hind water) since one week. She was a booked case with routine antenatal follow ups with no significant complaints. The routine ultrasound scans revealed no clue of any abnormality. She gave history of miscarriage in her first pregnancy due to multiple fetal abnormalities but 2^nd^ child was alive with normal health. The noteworthy finding was that the parents were paternal first cousins. The newborn was 2.9 Kg with APGAR 6/1, 8/5, no cyanosis, head circumference: 35.5cm, length of 48cm. General appearance was of thick erupted scaly skin separated by erythematous fissures extending deep into the dermis. Limbs were semi flexed with fused fingers and toes. There was peeling of skin all over the body. Eclabium, ectropium, flattening of ears and nose were noted with distorted facial features (Fig.1). All natural orifices were patent and vitals were normal. He was born with bilateral cryptorchidism. He had all other systems normal with soft non tender abdomen and normal vesicular breathing. After birth, baby’s skin started shedding off and on 4th day of birth his whole skin was peeled off (Fig.2). He was maintained in neonatal intensive care unit (NICU) and was given IV fluids only. Despite intensive care, oxygen therapy and IV fluids, the baby died on 11th day of birth in hospital. The parents were counseled and comforted. The possible causes of the disease was described and genetic counseling was advised for upcoming gestations.

## DISCUSSION

Harlequin ichthyosis; also labelled as ichthyosis congential or keratosis diffusa foetalis, is a tremendously infrequent genetic disorder with the incidence of one in 300,000 live births. HI exhibits no affinity towards any particular race or gender distribution however, fetuses of consanguineous marriages are prone to develop HI and this finding was obvious in the above mentioned case.^10^

Mutation in the ABC transporters ABCA12, a cell membrane transporter concomitant with lipid transportation are considered to be the underlying pathogenesis of HI. The newborns affected by this mutation have defective lipid secretion within epidermal keratinocytes resulting in loss of the skin lipid barrier and progression to HI. Chances of prolonged survival of newborns with HI are reliant on type of mutations. Homozygous mutations carry less chance of survival as compared to heterozygous ones.^8^,^9^ In present case, child survived for 10 days but there was no available facility of genetic testing to which would establish the type of mutation if present. Antenatal examination has a key role in the diagnosis of harelequin ichtyosis by exploration of amniotic fluid cells and an ultrasound mainly of the fetal mouth specifically at the 17^th^ week of pregnancy.^11^ Despite routine antenatal follow ups using ultrasound, HI was not diagnosed. Ultrasonography with color Doppler imaging is an imperative technique to diagnose HI. Since it is difficult to diagnose fetal HI, the HI genetic testing and counseling becomes imperative. Additionally a detailed account of family history, consanguinity, outcomes of previous pregnancies is essential. In this case detailed exploration of past history was done and intensive genetic counseling was arranged with mother.

Management of HI in the early neonatal period requires an integrated approach to avoid concomitant complications. Humidified incubator, thermoregulation, skin care and management of infectious should be taken care of in HI and same followed in current case. The retenoids use is treatment of choice due to its safety profile.^12^ Development of sepsis should be managed accordingly as it would remain the principal cause of mortality in neonates with HI. Furthermore the question to be raised is whether or not the 22-weeks anomaly scan could pick Harlequin fetus for early counselling during the antenatal follow ups.

## CONCLUSION

Harlequin icthyosis is a lethal condition of skin. Genetic counseling of parents is of vital importance especially in families with consanguineous marriages along with prenatal screening for mutated adenosine triphosphate binding-cassette transporter *ABCA12* gene in high risk patients for development of targeted therapy. Regardless of intensive care, the cases of HI cases do not terminate in pleasing outcome. Reliable strategies for early diagnosis of HI may decrease stress to family.

### Author`s Contribution

**JD and UK** conceived and wrote manuscript.

**ZR** did supervision, review, editing and final approval of manuscript.
